# e-Health, m-Health and healthier social media reform: the big scale view

**Published:** 2012-06-15

**Authors:** Yossi Bahagon, Orit Jacobson

**Affiliations:** Clalit Health Services, Israel; Clalit Health Services, Israel

**Keywords:** e-Health, mobile health, personal health record, online visit, patient empowerment, knowledge prescription

## Abstract

**Introduction:**

In the upcoming decade, digital platforms will be the backbone of a strategic revolution in the way medical services are provided, affecting both healthcare providers and patients. Digital-based patient-centered healthcare services allow patients to actively participate in managing their own care, in times of health as well as illness, using personally tailored interactive tools. Such empowerment is expected to increase patients’ willingness to adopt actions and lifestyles that promote health as well as improve follow-up and compliance with treatment in cases of chronic illness. Clalit Health Services (CHS) is the largest HMO in Israel and second largest world-wide. Through its 14 hospitals, 1300 primary and specialized clinics, and 650 pharmacies, CHS provides comprehensive medical care to the majority of Israel’s population (above 4 million members). CHS e-Health wing focuses on deepening patient involvement in managing health, through personalized digital interactive tools. Currently, CHS e-Health wing provides e-health services for 1.56 million unique patients monthly with 2.4 million interactions every month (August 2011). Successful implementation of e-Health solutions is not a sum of technology, innovation and health; rather it’s the expertise of tailoring knowledge and leadership capabilities in multidisciplinary areas: clinical, ethical, psychological, legal, comprehension of patient and medical team engagement etc. The Google Health case excellently demonstrates this point. On the other hand, our success with CHS is a demonstration that e-Health can be enrolled effectively and fast with huge benefits for both patients and medical teams, and with a robust business model.

**CHS e-Health core components:**

They include:

1. The personal health record layer (what the patient can see) presents patients with their own medical history as well as the medical history of their preadult children, including diagnoses, allergies, vaccinations, laboratory results with interpretations in layman’s terms, medications with clear, straightforward explanations regarding dosing instructions, important side effects, contraindications, such as lactation etc., and other important medical information. All personal e-Health services require identification and authorization.

2. The personal knowledge layer (what the patient should know) presents patients with personally tailored recommendations for preventative medicine and health promotion. For example, diabetic patients are push notified regarding their yearly eye exam. The various health recommendations include: occult blood testing, mammography, lipid profile etc. Each recommendation contains textual, visual and interactive content components in order to promote engagement and motivate the patient to actually change his health behaviour.

3. The personal health services layer (what the patient can do) enables patients to schedule clinic visits, order chronic prescriptions, e-consult their physician via secured e-mail, set SMS medication reminders, e-consult a pharmacist regarding personal medications. Consultants’ answers are sent securely to the patients’ personal mobile device.

On December 2009 CHS launched secured, web based, synchronous medical consultation via video conference. Currently 11,780 e-visits are performed monthly (May 2011). The medical encounter includes e-prescription and referral capabilities which are biometrically signed by the physician. On December 2010 CHS launched a unique mobile health platform, which is one of the most comprehensive personal m-Health applications world-wide. An essential advantage of mobile devices is their potential to bridge the digital divide. Currently, CHS m-Health platform is used by more than 45,000 unique users, with 75,000 laboratory results views/month, 1100 m-consultations/month and 9000 physician visit scheduling/month.

4. The Bio-Sensing layer (what physiological data the patient can populate) includes diagnostic means that allow remote physical examination, bio-sensors that broadcast various physiological measurements, and smart homecare devices, such as e-Pill boxes that gives seniors, patients and their caregivers the ability to stay at home and live life to its fullest. Monitored data is automatically transmitted to the patient’s Personal Health Record and to relevant medical personnel.

The monitoring layer is embedded in the chronic disease management platform, and in the interactive health promotion and wellness platform. It includes tailoring of consumer-oriented medical devices and service provided by various professional personnel—physicians, nurses, pharmacists, dieticians and more.

5. The Social layer (what the patient can share). Social media networks triggered an essential change at the humanity ‘genome’ level, yet to be further defined in the upcoming years. Social media has huge potential in promoting health as it combines fun, simple yet extraordinary user experience, and bio-social-feedback. There are two major challenges in leveraging health care through social networks:

a. Our personal health information is the cornerstone for personalizing healthier lifestyle, disease management and preventative medicine. We naturally see our personal health data as a super-private territory. So, how do we bring the power of our private health information, currently locked within our Personal Health Record, into social media networks without offending basic privacy issues?
b. Disease management and preventive medicine are currently neither considered ‘cool’ nor ‘fun’ or ‘potentially highly viral’ activities; yet, health is a major issue of everybody’s life. It seems like we are missing a crucial element with a huge potential in health behavioural change—the Fun Theory. Social media platforms comprehends user experience tools that potentially could break current misconception, and engage people in the daily task of taking better care of themselves.

CHS e-Health innovation team characterized several break-through applications in this unexplored territory within social media networks, fusing personal health and social media platforms without offending privacy. One of the most crucial issues regarding adoption of e-health and m-health platforms is change management. Being a ‘hot’ innovative ‘gadget’ is far from sufficient for changing health behaviours at the individual and population levels.

CHS health behaviour change management methodology includes 4 core elements:

1. Engaging two completely different populations: patients, and medical teams. e-Health applications must present true added value for both medical teams and patients, engaging them through understanding and assimilating “what’s really in it for me”. Medical teams are further subdivided into physicians, nurses, pharmacists and administrative personnel—each with their own driving incentive. Resistance to change is an obstacle in many fields but it is particularly true in the conservative health industry. To successfully manage a large scale persuasive process, we treat intra-organizational human resources as “Change Agents”. Harnessing the persuasive power of ~40,000 employees requires engaging them as the primary target group. Successful recruitment has the potential of converting each patient-medical team interaction into an exposure opportunity to the new era of participatory medicine via e-health and m-health channels.

2. Implementation waves: every group of digital health products that are released at the same time are seen as one project. Each implementation wave leverages the focus of the organization and target populations to a defined time span. There are three major and three minor implementation waves a year.

3. Change-Support Arrow: a structured infrastructure for every implementation wave. The sub-stages in this strategy include:

- Cross organizational mapping and identification of early adopters and stakeholders relevant to the implementation wave
- Mapping positive or negative perceptions and designing specific marketing approaches for the distinct target groups
- Intra and extra organizational marketing
- Conducting intensive training and presentation sessions for groups of implementers
- Running conflict-prevention activities, such as advanced tackling of potential union resistance
- Training change-agents with resistance-management behavioural techniques, focused intervention for specific incidents and for key opinion leaders
- Extensive presence in the clinics during the launch period, etc.

The entire process is monitored and managed continuously by a review team.

4. Closing Phase: each wave is analyzed and a “lessons-learned” session concludes the changes required in the modus operandi of the e-health project team.

